# Implementation of web-based hospital specialist consultations to improve quality and expediency of general practitioners’ care: a feasibility study

**DOI:** 10.1186/s12875-019-0960-5

**Published:** 2019-05-29

**Authors:** Thomas van der Velden, Bianca W. M. Schalk, Mirjam Harmsen, Guido Adriaansens, Tjard R. Schermer, Marc A. ten Dam

**Affiliations:** 1 Department of Primary and Community Care, Radboud University Medical Center, Nijmegen, the Netherlands; 2 Department of Primary and Community Care, Radboud University Medical Center, Radboudumc Transmural Knowledge and Innovation Center, postal route 117, PO Box 9101, 6500 HB Nijmegen, the Netherlands; 3 Scientific Center for Quality of Healthcare (IQ healthcare), Radboud University Medical Center, Nijmegen, the Netherlands; 4 Landelijke Huisartsen Vereniging, Nijmegen, the Netherlands; 5 Department of Internal Medicine, Canisius Wilhelmina Hospital, Nijmegen, the Netherlands

**Keywords:** Telemedicine, General practice, Primary health care, Remote consultation, Feasibility study

## Abstract

**Background:**

Rising healthcare costs due to unnecessary referrals to secondary healthcare services underscore the need for optimizing current referral procedures. This study investigates whether the use of web-based consultation (WBC) in general practice is a feasible alternative to decrease referrals.

**Methods:**

Patients with lumbosacral radicular syndrome, knee complaints, or thyroid dysfunction, who visited the general practitioner (GP) between May 2015 and December 2016 were included for a WBC. We determined whether the GP would refer a patient to an outpatient clinic in the absence of a WBC and then compared this decision with the referral advice from a specialist. We further assessed the user-friendliness of the WBC service based on average recorded user time and feedback from the GPs.

**Results:**

Seventy eligible WBCs submitted by GPs were analyzed. Our data showed a 46% absolute reduction in in-persons referrals in our study population. These findings confirmed the feasibility of using WBC. The median time spent to submit a WBC was five and 10 min for GPs and specialists respectively. On average, the WBC service saved €286 per WBC. The results of a questionnaire showed that GPs found WBC to be a user-friendly option which could help reduce the number of in-person referrals.

**Conclusion:**

We demonstrated that WBC is not only feasible but has the potential to reduce nearly half of all in-person referrals to outpatient clinics. WBC decreased healthcare expenses and proved to be a user-friendly and safe alternative to the standard referral process. WBC may potentially have a profound impact on healthcare expenditure if applied in a wider medical setting. For follow-up research, we recommend including a control group for comparative analyses.

**Electronic supplementary material:**

The online version of this article (10.1186/s12875-019-0960-5) contains supplementary material, which is available to authorized users.

## Background

Healthcare expenditure represents an increasingly substantial proportion of national costs in well-developed countries [1]. Hospital care largely contributes to these rising costs. In the Netherlands, access to more expensive specialized hospital care is only granted via referral by the general practitioner (GP). Consequently, GPs act as gatekeepers and have a pivotal role in containing these costs [2, 3].

Web-based consultation (WBC) with medical specialists has proven to be a less expensive and more patient-friendly alternative compared to an in-person referral to a hospital-based medical specialist [4]. In brief, WBC is a secure process which enables asynchronous communication between the GP and the specialist [5].

A recent systematic literature review concluded that WBC services are primarily focused on a single specialty, in particular dermatology or nephrology. However, in order for WBCs to have a greater impact on referral numbers, ideally GPs would be able to access a variety of specialists to assist in treatment options for a variety of medical conditions However, for the implementation of such a service and to maintain high quality, it would likely be most efficient to incorporate it into an existing system that is accessible by general practices in a specific geographical area [6].

For example, in the Ottawa-region (Canada), implementation of such an asynchronous WBC service was being studied in several medical specialties already, namely dermatology, internal medicine and neurology. This particular service provides GPs with easier and faster access to specialist knowledge when expert support is necessary, without the need for an in-person referral of the patient [7]. This system will likely be economically beneficial over the years, mainly due to a reduction in unnecessary referrals to outpatient clinics [8].

In their joint multi-annual projection planning, the Dutch Ministry of Health, Welfare and Sport, healthcare insurers and different healthcare parties agreed to reduce healthcare expenditure by reducing the number of referrals from GPs to outpatient clinics. They subsequently advised that alternative consultation methods, such as multi-specialty WBC services, should be developed and promoted [9]. Several studies in the Netherlands are currently evaluating WBC of medical specialists to determine the impact of WBC across different specialties. To date, tele-dermatology appears to decrease the number of in-person referrals, leading to efficient care at lower costs. Similar results have been found for tele-nephrology as well as for tele-pulmonology [5, 10, 11].

In this study we investigated whether the use of WBC can be expanded to more medical conditions. In order to be implementable in clinical settings, eligible conditions for WBC were selected under the assumption that the specialist would be able to answer the GP’s questions without face-to-face contact with the patient.

Our primary aim was to examine the feasibility of implementing a WBC service to reduce the number of in-person referrals for patients experiencing lumbosacral radicular syndrome, knee complaints, or thyroid dysfunction. Our secondary aim was to determine the user-friendliness and costs of WBC.

## Methods

### Setting

This prospective cohort study included WBCs of patients experiencing lumbosacral radicular syndrome, knee complaints, or thyroid dysfunction between May 2015 and December 2016. The study was initiated by GPs and specialists from the Canisius Wilhelmina Hospital (CWZ) in Nijmegen, a mid-sized secondary hospital situated in the southeastern part of the Netherlands. Approximately 250 GPs practicing in Nijmegen or the nearby vicinity, who primarily referred their patients to the CWZ, were offered the WBC service for patients with the aforementioned medical conditions in addition to the usual referral pathway or calling the specialist for advice. The opportunity to use WBC as alternative referral method was announced using an online advertisement as well as by oral presentations given at several general practices and regional meetings. The WBC service was incorporated in the existing Dutch electronic referral system called *ZorgDomein*, which is accessible by GPs and specialists [12]. Specialists had 3 days to respond after GPs initiated a WBC. Any available neurologist, internist or orthopedist with expertise in the relevant subject matter were able to respond to the WBC. Further contact between the GP and the specialist could occur, if required, for further clarification or if additional questions arose. The GP then informed the patient of the outcome of the WBC.

### Selection and consultation

The GP could initiate a WBC as an alternative to an in-person referral when expert medical consultation was needed for any patient who met the inclusion and exclusion criteria described in Table 1 and presenting with lumbosacral radicular syndrome, knee complaints, or thyroid dysfunction. The use of WBC as referral alternative was voluntary for the GP and patient. Lumbosacral radicular syndrome and knee complaints were selected because the hospital registry showed that a considerable proportion of these patients were referred back to the GP after only one or two visits to the outpatient clinic. Thyroid dysfunction was selected by a panel of specialists (comprising of internists) and GPs who considered that treatment decisions for this condition could be greatly facilitated by WBC. De-identified patient data were used.

**Table 1:** In- and exclusion criteria for patients suffering from lumbosacral radicular syndrome, thyroid dysfunction or knee complaints

Inclusion criteria	Exclusion criteria
Lumbosacral radicular syndrome	
- Age < 50 years with pain extension to one leg (sciatic pain)	- Onset of complaints > 50 year (age), continuously pain independent of position or movement, nocturnal pain, pain in both legs, widespread neurological paresis, micturition disorder (incontinence or retention), Cauda Equina syndrome
- And one or more of the following:	
○ Increase of pain in the leg during increase of pressure	
○ Pain and/or tingling corresponding to one dermatome: calf/lateral edge of the foot or instep/hallux	- Malaise, malignancy in medical history, unexplained weight loss, elevated erythrocyte sedimentation rate (ESR)
○ Positive test of Lasegue (pain past the knee when the straight leg is at an angle of less than 65 degrees)	- Long-term corticosteroids usage, length reduction, increased thoracic kyphosis
○ Finger-floor distance > 20 centimeterMotor deficits matching a segment	- Polyradiculopathy, elevated ESR
○ Absence of Achilles Reflex	- Second opinion
○ Complaints during < 6 weeks	
Knee complaints	
- Patient has (non-)traumatic knee complaints	None
Thyroid dysfunction	
- Overt hyperthyroidism or;	- Thyroid nodule
- Subclinical hyperthyroidism or;	- Overt hypothyroidism
- Subclinical hypothyroidism	

### Data collection

We collected data on patient characteristics from the WBC service database as well as the number of in-personal referrals within 2 months after their WBC from the hospital. Additional relevant information from GPs and specialists were also collected and included which course of action the GP would have taken had the WBC service not been available. The referral options of the GP were recorded as: 1) refer the patient to the specialist; 2) call the specialist; 3) treat the patient in primary care without referral or contacting the specialist. Based on the medical information provided by GPs, specialists described their advice and recorded whether they would advise the GP to: 1) refer the patient; 2) treat the patient in primary care. For each WBC, we investigated whether the patient had visited the hospital’s outpatient clinic for the medical condition within 2 months after the WBC in order to establish whether or not the patient had been referred for secondary care.

The user-friendliness of WBC was assessed by analyzing the average recorded time the GPs and specialists spent on each WBC and WBC submission time during the working day. General working hours were assumed to be between 8:00 am and 5:00 pm. In addition to information from WBC consultations, we assessed GPs’ experiences with the WBC service and reasons GPs declined to utilize the service using an online questionnaire in LimeSurvey® (Additional file 1). This survey was sent to all GPs in the study region and included a reminder to increase participation rates [13]. Open text comments on the service could be submitted in the questionnaire.

### Feasibility outcome and statistical analysis

Feasibility was determined by the influence of WBC on the number of in-person referrals. To investigate whether WBC reduced in-person referrals in our study population, we calculated the number of prevented referrals as the GP’s “refer the patient to the specialist” option combined with the specialist’s option “treat patient in primary care” minus the actual referrals within the 2 months follow-up period in that group. The proportional referral reduction was calculated as the prevented referrals divided by the GP’s number of intended referrals, including a continuity correction to approximate the binomial distribution. We repeated this calculation assuming that the referral option “call the specialist” was considered as GPs’ intention to refer to secondary care and these results were called “potential” referral reduction. The reduction in terms of referred patients was presented as a percentage with a 95% confidence interval (95% CI). User-friendliness, feedback from GPs on WBC, and reasons for not using the service were assessed using descriptive analyses. All analyses were done in SPSS (IBM SPSS Statistics for Windows, Version 22.0. Armonk, NY: IBM Corp).

### Cost analysis

Healthcare costs were estimated from the health insurance company’s (i.e. payer’s) and the patient’s perspectives. General costs from the payer’s perspective included service and consultation costs. Service costs included the use and maintenance of the electronic referral system (total yearly fee €250). Consultation costs were the fixed costs, which included the standard GP payment (€27), the standard specialist payment (€50), and fees associated with the electronic referral system (€10). Fixed prices for an outpatient clinic visit were derived according to the Dutch Diagnosis and Treatment Code Sets [in Dutch: *Diagnose Behandeling Combinatie (DBC)*]. Based on this information, average payments for lumbosacral radicular syndrome, knee complaints, and thyroid dysfunction were set at €572, €505, and €851 respectively and included associated costs for laboratory tests, medical imaging, follow-up consultations, and hospital fees.

Savings from the payer’s perspective were calculated separately for absolute referral reduction and potential referral reduction. The number of avoided referrals by medical condition was multiplied by the fee for an outpatient clinic visit. Benefits were defined as the costs subtracted from the savings.

Costs for the patient were €50 for the WBC and an additional fee in case of in-person referral to the outpatient clinic. For patients insured in the Netherlands, the maximum annual deductible for services outside of GP consultations was €385 in 2016. Thus, for the purposes of this study, the maximum patient’s contribution for an outpatient clinic consultation was €385.

## Results

**Fig. 1:**
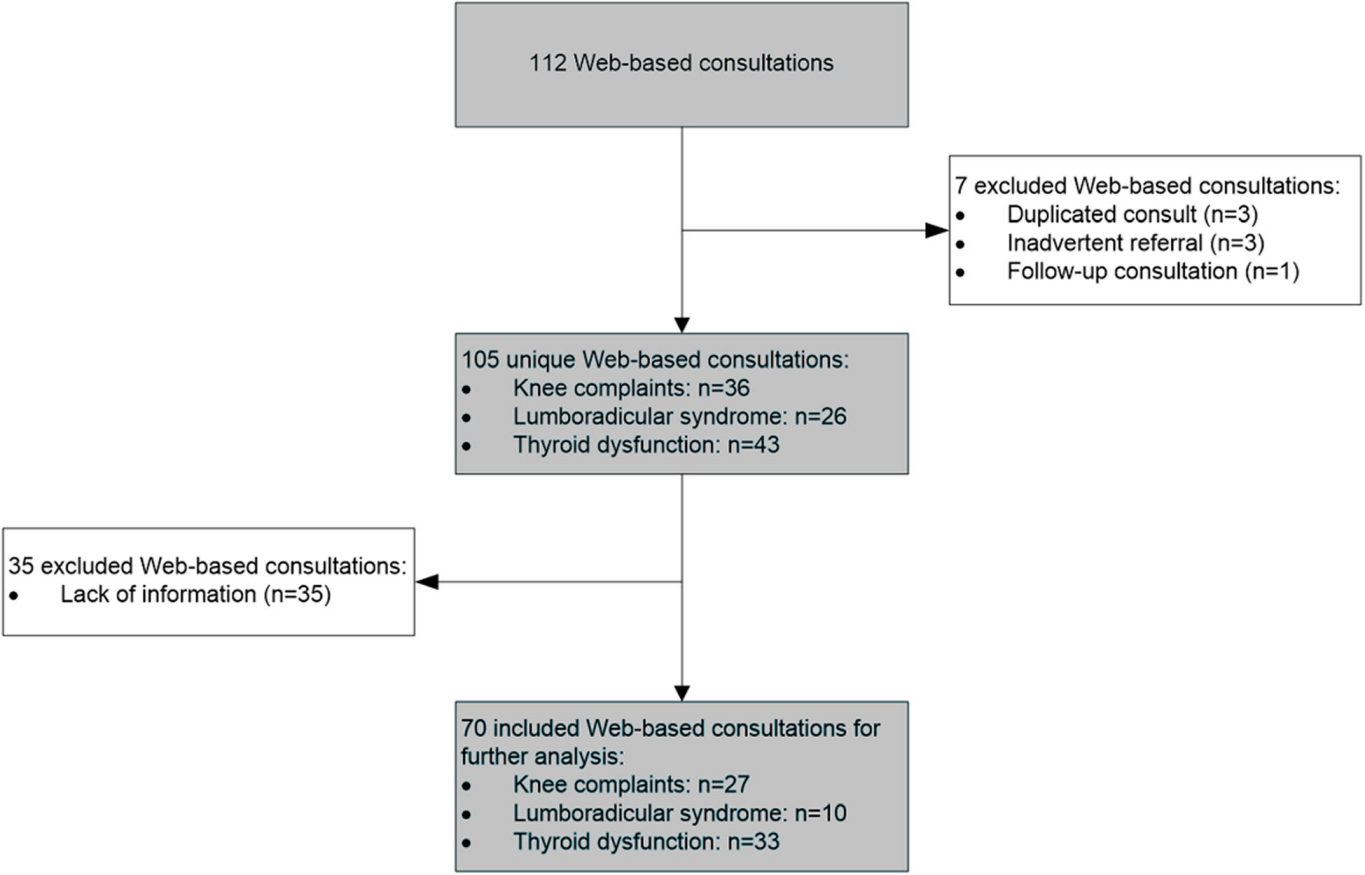
Flowchart of the selection of the study population and Web-based consultations between May 2015 and December 2016

During the study period, a total of 112 WBCs with a response from one of the hospital specialists were conducted. These WBCs were submitted by 67 GPs from 46 general practices. However, 42 WBCs were excluded due to an inadvertent referral (i.e. referral to another hospital or other region) (*n* = 3), missing information (*n* = 35), a follow-up consultation (*n* = 1), or because patients had accidentally been submitted twice by the GP (*n* = 3)(Fig. 1). Of the remaining 70 WBCs, the majority were handled by internists (*n* = 33, 47.1%), followed by orthopedists (*n* = 27, 38.6%), and neurologists (*n* = 10, 14.3%), and. The mean age of the patients was 55.9 (SD = 18.8) years and roughly one-third (*n* = 22, 31.4%) of the patients in the analysis were men (Table 2). Whereas most (87%) of the WBCs received a response by a specialist within 3 days, the remaining WBCs (13%) did not receive a response in this timeframe due to technical issues from the onset of the study. This response lag led to delayed treatment for a pregnant woman with hypothyroidism.

**Table 2:** Number of Web-based consultations, patient characteristics and the time investment

**Characteristics**	Lumbosacral radicular syndrome	Thyroid dysfunction	Knee complaints	Total
Demographics				
WBCs^a^, n (%)	10 (14.3)	33 (47.1)	27 (38.6)	70 (100)
Age in years, mean (SD^b^)	53.8 (13.2)	57.3 (20.6)	55.0 (18.8)	55.9 (18.8)
Male sex, n (%)	3 (30)	3 (9.1)	16 (59.3)	22 (31.4)
Time investment in minutes, median (p.25 - p.75)* [n]				
General Practitioners	5 (5–7.5) [*n* = 9]	5 (5–10) [*n* = 33]	5 (4.25–9.5) [*n* = 20]	5 (5–10) [*n* = 62]
Specialists	5 (5–7.75) [*n* = 10]	10 (5–10) [*n* = 19]	10 (10–10) [*n* = 25]	10 (5–10) [*n* = 54]

^a^WBCs: Web-based consultations

^b^SD: Standard Deviation

*p.25: 25th percentile. p.75: 75th percentile

Note: 70 eligible WBCs, performed between May 2015 and December 2016 were analyzed

### User-friendliness

The median time spent to submit a WBC was five and 10 min for GPs and specialists respectively (Table 2). Time of submission was registered in 58 WBCs, 69% of the WBCs were submitted during general working hours.

### Reduction of referrals

The referral options of the GPs were compared to the referral advice of the specialists. In the hypothetical absence of a WBC, GPs reported that they would have referred 28 patients; notably, in 16 out of these 28 cases, specialists did not advise a referral in the WBC. Nonetheless, three of these 16 patients still visited the outpatient clinic within two months. Thus, WBC reduced the total number of referrals to secondary care from 28 to 13 patients (Table 3), translating to a 46% (95% CI: 28–66%) absolute reduction in referrals. The contrast was seen in eight cases, i.e. specialists advised referral to the outpatient clinic, but the patients did not visit this particular outpatient clinic during the 2-month follow-up period (Table 3). When the referral option “call the specialist”, was added as an option, the total number of potential referrals was reduced from 63 to 37 referrals, a 59% (95% CI: 46–71%) absolute potential reduction in referrals (Table 3). These findings confirmed the feasibility of using WBC to reduce the number of in-person referrals. Overall, 27 of the 70 (38.6, 95% CI: 27–51%) WBCs were followed-up by an in-person referral to the specialist. For patients with thyroid disease in particular, the specialist advised the GP to take further steps, such as initiating the treatment course, while the patient waited for an in-person consultation with the specialist.

**Table 3:** General practitioner’s referral option compared to the specialist’s advice in the Web-based consultation and the (potential) referral reduction per medical condition

	Specialist’s referral advice						
General practitioner’s referral option	Overall [*n* = 70]	Refer patient [*n* = 20]			Treat patient in primary care [*n* = 50]		
		Total	OPC^b^ visit	No OPC^b^ visit	Total	OPC^b^ visit	No OPC^b^ visit
Refer patient	28	12	9	3	16	3	13
Call the specialist	35	7	2	5	28^a^	3	24
Treat patient in primary care	7	1	1	0	6	1	5
	Lumbosacral radicular syndrome		Thyroid dysfunction		Knee complaints		Total
Referral reduction per medical condition (95% CI^c^)^d^	100% (20–100)		38% (10–74)		44% (22–69)		46% (28–66)^d^
Potential referral reduction per medical condition (95% CI^c^)	57% (20–88)		69% (50–83)		46% (26–67)		59% (46–71)

^a^Unknown whether or not one of the patients visited the outpatient department within the 2 months follow-up period

^b^OPC: Outpatient clinic: the patient visited /did not visit the OPC within the 2 months follow-up period

^c^95% CI: 95% Confidence Interval

^d^This referral reduction was calculated as follows (see line “Refer Patient”): (total number of treat patient in primary care by specialist – OPC visit) / overall number in the line “refer Patient” = (16–3)/28*100%

### Costs and savings

The costs from the payer’s perspective per WBC were €93, including GP and specialist consultation costs as well as service costs related to the electronic referral system. The costs of the 28 intended referrals were €2603. Additional costs, generated by seven cases in which GPs wanted to treat the patient in primary care were €651. The absolute referral reduction for the 13 patients saved €7727. The absolute benefit for the payer was €4474. The costs of the 35 intended calls to the specialist were €3254. The potential referral reduction of 24 patients saved €18,808. The potential additional benefit for the payer was €15,554. Overall, the WBC service potentially saved €20,027 (i.e. €286 per WBC).

In general, the benefit for patients was €335 per avoided referral. Six WBCs were unnecessary, since the GP and specialist both wanted to treat the respective patients in primary care, which cost the patient €50.

### GPs’ experiences with WBC

The WBC evaluation questionnaire was completed by 38 GPs of whom 19 used WBC. The majority of the GPs agreed with most of the statements stated in the questionnaire (Table 4). Reasons for not using the service were as follows: seven GPs missed the advertisement letter of the possibility to use WBC or did not implement it in their daily routine, six GPs had no eligible patients during the intervention period, and three GPs did not view WBC as a better alternative for referral when compared to consulting a specialist by telephone. Other reasons for not using the WBC service were preference to refer directly to the specialist, the habit to refer or contact specialists in other hospitals, and unfamiliarity with the added value of WBC.

**Table 4:** Feedback of general practitioners on the Web-based consultation service

Percentage of the general practitioners who ‘agreed’ or ‘completely agreed’ to the statement	Users of WBC^a^[*n* = 19]
The WBC^a^ service is user friendly	95%
WBC^a^ contributes to avoid unnecessary referrals	90%
WBC^a^ contributes to my knowledge of the specific complaint	63%
WBC^a^ is a good alternative for referring to outpatient clinics	58%
WBC^a^ is an improvement compared to consulting a specialist by phone	74%
The specialist’s response through WBC^a^ was helpful	90%
I am satisfied with the specialist’s response time to the WBC^a^	100%

^a^*WBC* Web-based consultation

## Discussion

### Main findings

The results of this study support the feasibility of a regional WBC service as an alternative referral method to gain access to medical specialists’ expert advice. Strikingly, WBC reduced the number of in-person referrals by nearly half, reducing healthcare expenses over the study period, while remaining a user-friendly and safe alternative. Overall, GPs’ feedback on the user-friendliness of the WBC service and the potential to avoid unnecessary referrals was resoundingly positive.

### Comparison with existing literature

The absolute reduction of in-person referrals with WBC in our study (46%) is comparable with previous findings in the international literature. For instance, Keely et al. observed a 43% referral reduction in Canada wherein GPs could consult any medical specialist [7]. Tele-nephrology reduced 60% of all referrals in a Dutch study of *Scherpbier* et al.^5^, whereas tele-dermatology led to an even larger reduction of 68% of GP referrals when the GP intended to refer [10]. The WBC service in our study was time efficient with rapid response times from specialists similar to earlier research from the Netherlands and Canada [5, 7]. However the €286 cost containment per WBC for the healthcare payer was markedly higher compared to the €10 cost containment reported by *Liddy* et al. [8] A possible explanation for this difference may be due to additional referral costs. They included the extra costs in cases where the GP chose not to refer the patient but the specialist did after WBC [8]. One limitation of our cost analysis is that we did not estimate other potential cost savings from the payer’s and the patient’s perspective due to WBC, such as earlier access to specialty care resulting in quicker treatment, fewer travel expenses and less work absenteeism. [8] In earlier studies, GPs have reported high satisfaction with WBC services, which is in line with the feedback received from the GPs in our study. One of the reported advantages we did not investigate in our study is the educational effect of WBC. Initial increased contact with specialists via WBC can reduce the number of WBCs or referrals in the long term as GPs become more knowledgeable of the conditions [14, 15].

### Strengths and limitations

One of the major strengths of this study is that it is one of the first studies to investigate the feasibility of a WBC service in a usual care setting, without selection of GPs or specialists. The WBC service was implemented in an existing referral service accessible by GPs who primarily referred their patients to hospital for secondary care. Moreover, we provided a detailed overview of the benefits by estimating the costs and potential savings from the healthcare payer’s and patient’s perspectives. We further assessed GPs’ experiences with the WBC service to improve implementation of this service in the future. Together, these aspects provider a comprehensive understanding of WBCs to better inform health care providers and policy makers.

The small study population is one of the limitations in this study. This is partly attributed to obstacles at the onset of the project, which led to a lower number of included patients than expected and incomplete data collection for analysis. Furthermore, some GPs in the region may have declined to participate since they were unfamiliar with the WBC service. It is also important to note that despite examining only three medical conditions, the percentage of avoided referrals is comparable to previous studies investigating multiple specialties over a similar study period [7]. Furthermore, we identified eight cases in which the specialists advised referral to an outpatient clinic, but the patients did not attend. We did not collect reasons for non-attendance. Whereas this number did not affect the referral reduction and the costs, delayed intervention could potentially increase long-term costs. Patient preference on when to visit the hospital and which hospital might be a possible explanation for non-attendance within the two-month period. Given the study design, there is no control group to compare referrals by GPs that did not use WBC. In addition, we did not collect any information on characteristics of GPs using WBC and their practices. Lastly, caution should be taken when extrapolating our results to the Dutch population, since only one hospital was involved in our study.

### Implications for research and/or practice

Our feasibility analysis supports widening the scope of WBC by applying it across more medical disciplines, which could ultimately improve timely access to specialized care and fewer unnecessary hospital visits for patients. In cases where an in-person referral is required, waiting lists to access outpatient clinics would have been reduced, and the specialist would be able to initiate diagnostics or therapy via the GP without delay. For example, in our study, several GPs initiated treatment for patients with thyroid dysfunction upon WBC with the specialist.

For follow-up research, we emphasize the importance of having a comprehensive overview of the flow between the GPs, their practices and the patient. We recommend that the entire process is described, beginning with inclusion of the GP and their practices, the patient follow-up, the specialist feedback and the subsequent follow-up based on this feedback. Further recommendations are to extend the study period, include a control group for comparative analyses, broaden the WBC service to include additional medical conditions /specialties, and to expand the service to a larger area within the Netherlands. Furthermore, to provide insight into the use of WBC, data collection would ideally include all information in the care process and reasons for non-attendance when specialists’ advice is “refer patient”, thus providing a complete view from the general practice’s point of view. To increase the chance of success, it would be also beneficial to assess the needs of GPs regarding the expansion to specific medical conditions/specialties as well as the educational effect of WBC on GPs. Implementation strategies to promote the use of WBC among GPs should be continued to increase the availability of WBCs. It is also essential to evaluate patients’ satisfaction and specialists’ feedback from the service since this information can drive further improvements to the WBC service.

## Conclusions

We demonstrated that WBC is a feasible service to implement. It has the potential to reduce nearly half of in-person referrals to outpatient clinics for patients with lumbosacral radicular syndrome, knee complaints, and thyroid dysfunction. Based on our study, this reduction in in-person referrals resulted in a marked decrease in healthcare expenditure. The service appears to be a user-friendly and safe system when implemented correctly. We anticipate increasing use of WBCs in the future as the service gains more publicity.

## Additional file

Additional file 1: Questionnaire to assess experience of general practitioners with the Web-based consultation service and reasons for not using the service. (DOCX 29 kb)

## Abbreviations

CI: Confidence interval
CWZ: Canisius Wilhelmina Hospital
GP: General practitioner
KC: Knee complaints
LHV: Dutch Association of General Practitioners
LRS: Lumbosacral radicular syndrome
SD: Standard deviation
SPSS: Statistical package of social science
TD: Thyroid dysfunction
WBC: Web-based consultation
