# Supplementary material for: Implementation of web-based hospital specialist consultations to improve quality and expediency of general practitioners’ care: a feasibility study
Source: BMC Fam Pract. 2019 May 29;20:73. doi: 10.1186/s12875-019-0960-5 (PMC6540440; doi:10.1186/s12875-019-0960-5)
Supplement: Supplementary file 1 — Questionnaire to assess experience of general practitioners with the Web-based consultation service and reasons for not using the service. (DOCX 29 kb) [file 12875_2019_960_MOESM1_ESM.docx]

**Appendix I. Questionnaire to assess experience of general practitioners with the Web-based consultation service and reasons for not using the service**

**General practitioners that used the service**

*Use the scale below to indicate the degree to which you agree with the following statements.*

S1: The Web-based consultation service is user friendly:

Completely disagree 1 2 3 4 5 Completely agree

S2: Web-based consultation contributes to avoid unnecessary referrals:

Completely disagree 1 2 3 4 5 Completely agree

S3: Web-based consultation contributes to my knowledge of the specific complaint:

Completely disagree 1 2 3 4 5 Completely agree

S4: Web-based consultation is a good alternative for referring to the outpatient department:

Completely disagree 1 2 3 4 5 Completely agree

S5: Web-based consultation is an improvement compared to consulting a specialist by phone:

Completely disagree 1 2 3 4 5 Completely agree

S6: The specialist’s response through Web-based consultation was helpful:

Completely disagree 1 2 3 4 5 Completely agree

S7: I am satisfied with the specialist’s response time to the Web-based consult:

Completely disagree 1 2 3 4 5 Completely agree

**General practitioners that did not use the service**

Q1: What is the reason that you did not use Web-based consultation (more answers possible)

- No eligible patients during the intervention period
- I was not notified of the possibility to use Web-based consultation
- Web-based consultation is not user friendly
- I’d rather refer my patients directly to the specialist
- Web-based consultation is no good alternative for referring compared to consulting a specialist by phone for me as general practitioner
- Web-based consultation is no good alternative for referring compared to consulting a specialist by phone for my patients
- Other (please explain)

**All general practitioners**

Q2: Have you experienced troubles or do you have suggestions to improve Web-based consultation?

Q3: Additional feedback concerning Web-based consultation:

S:statement. Q: question.
